# The inhibitory effect of melatonin on osteoclastogenesis of RAW 264.7 cells in low concentrations of RANKL and MCSF

**DOI:** 10.3906/biy-2007-85

**Published:** 2020-12-14

**Authors:** Hala JARRAR, Damla ÇETİN ALTINDAL, Menemşe GÜMÜŞDERELİOĞLU

**Affiliations:** 1 Bioengineering Division, Department of Chemical Engineering, Faculty of Engineering, Hacettepe University, Ankara Turkey

**Keywords:** RAW 264.7, melatonin, RANKL, MCSF, osteoclast differentiation

## Abstract

RAW 264.7 cells are one of the most recommended cell lines for investigating the activity and differentiation of osteoclasts. These cells differentiate into osteoclasts in the presence of two critical components: receptor activator of nuclear factor kappa B ligand (RANKL) and macrophage colony stimulating factor (MCSF). Melatonin (MEL) hormone has recently become one of the small molecules used in the field of bone regeneration and bone disease treatment, as it has the ability to inhibit the differentiation of osteoclasts directly by suppression of the NF-κB signaling pathway. The main aim of the current study is to determine sufficient RANKL/MCSF concentrations for differentiation of the cells to osteoclasts and to describe the repressive effect of MEL on the osteoclastogenesis of these cells. In this regard, it was found that 10 ng/mL of RANKL- and MCSF-containing medium is suitable for inducing osteoclastogenesis of the cells. In addition, melatonin at doses in the range of 100–1000 µM does not have a cytotoxic effect. Subsequently, results of tartrate resistant acid phosphatase (TRAP) activity, TRAP staining, and relative expressions of cathepsin K, nuclear factor of activated T cells one (NFATC1), and TRAP genes showed a suppressive effect of MEL —especially 800 µM— on RANKL-induced osteoclastogenesis of these cells.

## 1. Introduction

Osteoclasts are responsible for bone resorption in a defect area, which is subsequently followed by bone building mediated by osteoblasts (Ai-Aql et al., 2008). Osteoclast dysfunction may cause bone diseases such as osteopetrosis and osteoporosis (Nguyen and Nohe, 2017). Osteoclast formation is regulated directly and indirectly by three main factors including macrophage colony stimulating factor (MCSF), osteoprotegerin (OPG), and receptor activator of nuclear factor kappa B ligand (RANKL) (Ai-Aql et al., 2008). The attraction between RANKL and its receptor (RANK) initiates the differentiation process, and interaction between them is controlled by an antagonist factor called OPG (Kholi and Kholi, 2011). Meanwhile, MCSF facilitates proliferation of osteoclast precursors and the survival of osteoclasts (Fan et al., 1997).

Bone marrow macrophages (BMMs) were used to obtain osteoclasts; however, it is difficult to isolate and maintain this primary culture and obtain a homogenous population of precursors. Furthermore, they require additional nutrients. As an alternative, the RAW 264.7 cells became a significant tool for studying the activity and differentiation of osteoclasts because they have many advantages over BMM cells such as easy culturing and passaging, widespread availability, and the homogeneous nature of the osteoclast precursor populations (Nguyen and Nohe, 2017; Kong et al., 2019). RAW 264.7 is a macrophage/monocyte cell line which originated from Abelson murine leukemia virus-induced tumor in *Mus musculus* mice (Kong et al., 2019). These cells have the ability to differentiate toward osteoclasts in the presence of RANKL. Furthermore, the addition of MCSF to the osteoclastogenic medium supports the proliferation of osteoclasts. However, the cited concentrations and combinations of both factors vary in the literature. For instance, many studies have used RANKL alone in the osteoclastogenic medium, while others have used both RANKL and MCSF. Moreover, different concentrations of each factor in the range of 10–100 ng/mL were mostly supplemented. Additionally, some of the studies used equal concentrations of each factor, while others used different concentrations of each (Hirotani et al., 2004; Ichikawa et al., 2006; Xu et al., 2009; Ghayor et al., 2011; Germaini et al., 2017).

Melatonin (MEL) is a vital hormone synthesized by the pineal gland of vertebrates. In addition to its well-known functions in the modulation of the sleep/awake cycle and circadian and seasonal rhythms, it has been used as an anticancer and antioxidant agent (Kaplan et al., 2015; Çetin Altındal and Gümüşderelioğlu, 2019). Furthermore, MEL exerts a significant effect on bone remodeling by suppressing bone resorption and promoting bone regeneration through a decrease in osteoclast activity and an increase in osteoblast activity, respectively (Liu et al., 2013). For that reason, MEL can be used to treat osteoporosis where osteoclast activity is high (Wang et al., 2015). The repressive effects of MEL on osteoclast activity were either direct or indirect. Indirect inhibition is an enhancement of OPG secretions by osteoblasts which in turn antagonizes RANKL binding by preventing osteoclast differentiation (Koyama et al., 2002). In addition, MEL doses within the range of 100–500 µM were found to have a direct suppressive effect on the differentiation of osteoclast precursors (Kim et al., 2012; Zhou et al., 2017).

However, the importance of different MEL concentrations on osteoclastogenic inhibition of RAW 264.7 cells was not reported in the literature; most studies have used BMMs. Moreover, high concentrations of RANKL and MCSF were used in differentiation studies of macrophages. In this paper, the aim is to investigate the differentiation capacity of RAW 264.7 cells toward osteoclasts in proliferation medium and osteoclastogenic medium by using concentrations of RANKL and MCSF that are relatively lower than those reported in the literature. Additionally, we investigate the direct inhibitory effect of different MEL concentrations (500 µM and 800 µM) on differentiated cells.

## 2. Materials and methods

### 2.1. Materials

RAW 264.7 cells (ATCC® TIB-71TM) were obtained from the American Type Culture Collection (ATCC). Melatonin, RANKL, and MCSF were obtained from Sigma–Aldrich Chemie GmbH (Hamburg, Germany) and L-glutamine, fetal bovine serum (FBS), penicillin–streptomycin, and Dulbecco’s modified eagle medium (DMEM) were bought from Capricorn Scientific GmbH (Ebsdorfergrund, Germany). 3-[4,5-Dimethylthiazol-2-yl]-diphenyltetrazolium bromide (MTT) and isopropanol were purchased from Sigma–Aldrich Chemie GmbH. Diamidino-2-phenylindole (DAPI) and Alexa Fluor 488 phalloidin were obtained from Invitrogen (Thermo Fisher Scientific Inc., Waltham, MA, USA). For TRAP activity studies, p-nitrophenyl phosphate tablets (PNPP) and iron chloride hexahydrate were obtained from Sigma–Aldrich Chemie GmbH, and disodium tartrate dehydrate was purchased from Merck KGaA (Darmstadt, Germany). Naphthol AS MX phosphate and fast red violet LB salt were obtained from Sigma–Aldrich Chemie GmbH, sodium acetate anhydrous was obtained from Honeywell Riedel-de Haën AG (Seelze, Germany), and N, N-dimethylformamide was purchased from Merck KGaA for TRAP staining.

### 2.2. Characterization of the cells

The cells were seeded in proliferation medium (high-glucose–containing DMEM 10% v/v, FBS 2% v/v, L-glutamine and 1% v/v penicillin–streptomycin; inoculation density 1 × 104 cells/mL) in tissue culture polystyrene (TCPS) dishes. The culture continued for 12 days without changing the medium.

#### 2.2.1. Cell viability and cell counting

Mitochondrial activities of the cells were determined by MTT studies. Six hundred μL of serum-free medium with 60 μL of MTT reagent were placed in the wells. Then, they were incubated at 37 °C in a CO2 incubator for 3 h. The resulting formazan crystals were dissolved by 400 μL of isopropanol solution containing 0.04 M of hydrochloric acid. The absorbance was determined at 570 nm wavelength (reference 690 nm) by plate reader (Double Beam UV-visible spectrophotometry; Labomed, Inc., Los Angeles, CA, USA).

For the determination of living cell number, cells were separated from the surface by cell scrapper, and living cells in the cell suspension were counted by hemocytometer (Neubauer chamber).

#### 2.2.2. SEM analysis

Morphology of cells was observed by SEM. At predetermined times, cell fixation was performed by incubation in glutaraldehyde solution (2.5%) for 30 min. Then, the cells were exposed to an ethanol series (10%–100%) for dehydration. Afterwards, the samples were incubated for 5 min in hexamethyldisilazane (HMDS) and dried overnight. Samples were finally coated with gold–palladium, and imaging was performed at different magnifications (FIB-SEM, TESCAN, GAIA 3, Czech Republic).

### 2.3. Cytotoxic effect of melatonin on the cells

The effect of different melatonin concentrations (0–1000 µM) on proliferation of the cells was initially determined to ensure that inhibition of osteoclast formation is caused by the melatonin inhibitory effect and not by cytotoxic effect. For this purpose, different melatonin concentrations were prepared in proliferation medium. After 24 h, prepared melatonin solutions were added, and the MTT study was performed as mentioned in Section 2.2.1.

### 2.4. Determination of osteoclastogenic medium composition

For determination of osteoclastogenic medium components that are able to promote osteoclast precursor differentiation towards osteoclasts, a comparative study was performed by cultivating RAW 264.7 cells in three different media including proliferation medium, proliferation medium containing 10 ng/mL RANKL, and proliferation medium containing 10 ng/mL RANKL + 10 ng/mL MCSF. Media were replenished every 2–3 days. Cell viability in the different groups was determined by MTT assay as mentioned in Section 2.2.1.

#### 2.4.1. TRAP activity and TRAP staining

The TRAP enzyme synthesized by osteoclasts was detected qualitatively by staining and quantitatively by enzyme activity assay. TRAP activity was detected by determining the absorbance resulting from conversion of p-nitrophenol to p-nitrophenolate. Consequently, cells were lysed by adding 200 μL of Triton X-100 solution (0.1%) to each sample and sonicated at 4 °C. After centrifugation at 13,000 rpm, 20 μL of supernatant was added to 180 μL of TRAP solution (2.5 mM pNPP; 0.1 M sodium acetate buffer, pH 5.8; 0.2 M KCl; 10 mM disodium tartrate; 1 mM ascorbic acid; and 100 μM FeCl3) and incubated at 37 °C for 30 min. Lastly, absorbance values were measured by plate reader at 405 nm.

For TRAP staining, the cells were incubated in formaldehyde solution (4%) at 4 °C for 10 min and acetone–ethanol (1:1) solution for 1 min. Samples were finally washed thrice with PBS and kept at 4 °C. Previously fixed samples were subsequently stained by TRAP staining solution. This solution was prepared with staining buffer which consisted of 40 mM of sodium acetate and 10 mM of disodium tartrate dehydrate (pH 5). Next, the final staining solution was obtained by adding 0.01% (w/v) naphthol AS-MX phosphate, 0.06% (w/v) fast red violet LB salt, and 1% (v/v) N, N-dimethylformamide to the staining buffer. Then, the cells were kept in staining solution at 37 °C for 1 h. After washing the cells with PBS, imaging was performed by light microscope.

#### 2.4.2. DAPI/F-actin staining

The nuclei and cytoskeletons of the cells grown in TCPS were observed by DAPI/F-actin staining. The cells were exposed to 4% (v/v) formaldehyde solution for 20 min to fix the cells and Triton X-100 (0.1%) solution for 10 min to increase the permeability of the cell membrane. Filamentous actins of the cells (F-actin) were stained with 1% (v/v) Alexa Fluor 488 phalloidin solution in PBS, while the nuclei of the cells were stained with 0.1% (v/v) DAPI solution in the dark for 30 min. Samples were imaged using fluorescence microscopy (Olympus IX71; Olympus America Inc., Center Valley, PA, USA).

#### 2.4.3. RT-PCR analysis

RT-PCR analysis was performed to determine relative expressions of cathepsin K, TRAP, and NFATC1. Full-length RNA was extracted using RNeasy mini kit (Qiagen GmbH, Düsseldorf, Germany) from the samples collected in Qiazol (Qiagen GmbH) at the desired time intervals. Briefly, 125 µL of chloroform was added to Qiazol-containing samples and shaken 10–15 times. Then, the samples were centrifuged at 4 °C and 13,000 rpm for 10 min. The top part of the solution was transferred to new RNA-DNA free Eppendorf tubes and mixed with the same amount of 70% (v/v) ethanol solution. The mixture was transferred to RNeasy spin columns containing RNA-selective membranes and centrifuged at 13,000 rpm for 1 min. Liquid from the bottom was removed, and a 700 µL RW1 buffer solution was added to each well and centrifuged at 13,000 rpm for 30 s. After this step, a 500 µL RPE buffer solution was added to the columns and centrifuged twice. As a final step, 30 µL RNase-free water was added, and centrifugation was performed for 1 min. Isolated RNA concentration was determined by Nanodrop 2000 (Thermo Fisher Scientific Inc.). Then, cDNA was synthesized using a high-capacity cDNA kit (Applied Biosystems, USA). Relative expressions of the targeted genes were determined by LightCycler® Nano (F. Hoffman–La Roche Ltd., Basel, Switzerland) using 5X HOT FIREPol®EvaGreen®qPCR Supermix and normalized to the housekeeping gene β-actin. Obtained data were analyzed using the 2-(ΔΔCt) method. The group containing cells grown in proliferation medium on day 5 was selected as the control group. Gene expression of the other groups was calculated compared to the control. The reverse and forward primer sequences are given in Table.

**Table:** Table. Primers used in RT-PCR analysis of osteoclast marker genes.

Gene	Forward primer 5’-3’	Revers primer 5’-3’
β-actin	GTGCTATGTTGCCCTAGACTTCG	GATGCCACAGGATTCCATACCC
TRAP	AGATTTGTGGCTGTGGGCGA	CTGCACGGTTCTGGCGATCT
NFATC1	GGGTCAGTGTGACCGAAGAT	GGAAGTCAGAAGTGGGTGGA
Cathepsin K	CACCCAGTGGGAGCTATGGAA	GCCTCCAGGTTATGGGCAGA

### 2.5. Determination of the inhibitory dose of melatonin

Two nontoxic concentrations (500 µM and 800 µM) were selected and used to investigate inhibitory effects on osteoclastogenesis of the cells. The control group was 0 μM melatonin-containing differentiation medium (proliferation medium containing 10 ng/mL RANKL and 10 ng/mL MCSF). The two abovementioned concentrations were prepared, and the cells were seeded as mentioned in Section 2.3. On day 5, variations in cell viability among the different groups were assessed by MTT analysis as described in Section 2.2.1. Furthermore, TRAP activity and TRAP staining were performed as mentioned in Section 2.4.1. The relative expressions of osteoclast marker genes were also determined by RT-PCR as shown in Section 2.4.3.

### 2.6. Statistical analysis

The experimental studies were performed with three parallels, and GraphPad InStat software (GraphPad Software Inc., La Jolla, CA, USA) was used for statistical analysis. In order to determine statistical differences among the groups, one-way ANOVA was used with P > 0.05 representing no significant differences.

## 3. Results

### 3.1. Characterization of the cells

**Figure 1:**
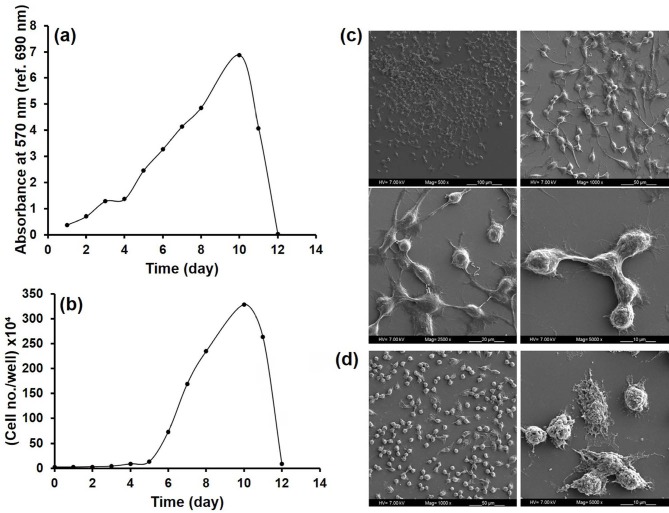
Characterization of RAW 264.7 cells; (a) MTT graph, (b) graph of hemocytometric counting, (c) SEM images of cells on day 6 (scale bars represent 100 μm, 50 μm, 20 μm, and 10 μm), (d) SEM images of cells on day 12 (scale bars represent 50 μm and 10 μm).

The absorbance values obtained from MTT analysis and cell numbers obtained by hemocytometric counting during 12 days of culture were plotted versus time (Figures 1a and 1b). Lag phase, log phase and the stationary and death phases are observed in the growth curves. Cells entered into the log phase on the 5th day of culture. The specific growth rate of the RAW 264.7 cells was calculated as 0.045 h–1, and doubling time was 15 h. SEM images were first taken on the 6th day of culture, and it was determined that cells attached to the TCPS surface successfully and connections among them were established (Figure 1c). In addition, the cells have both spindle-shaped and rounded morphologies. Figure 1d also shows that the connections among cells were disrupted as cells entered the death phase on the 12th day.

### 3.2. Cytotoxicity of melatonin

**Figure 2:**
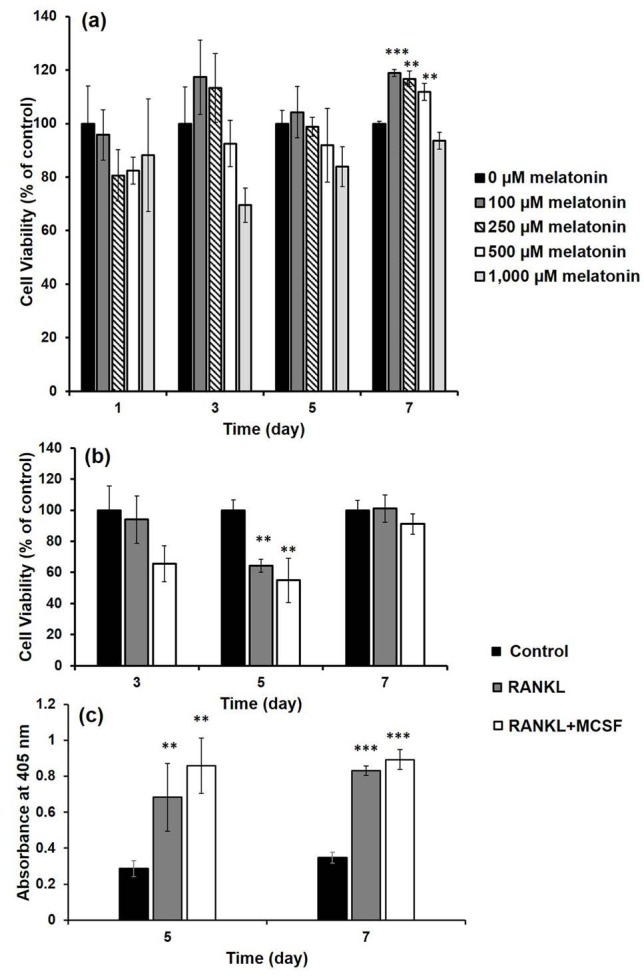
a) MTT results of RAW 264.7 cells grown in proliferation medium supplemented with melatonin concentrations in range of 0–1000 μM (Statistical differences when melatonin-free group is control: * P < 0.05, ** P < 0.01, *** P < 0.001), b) Cell viability, and c) TRAP activity of RAW 264.7 cells cultured in different media. (Control: cells in proliferation medium; RANKL: cells in proliferation medium containing 10 ng/mL RANKL; RANKL+MCSF: cells in proliferation medium containing 10 ng/mL RANKL and 10 ng/mL MCSF). (Statistical differences according to control group: * P < 0.05, **P < 0.01, *** P < 0.001).

The viability of the cells was maintained during the culture period at all concentrations of melatonin as seen in the MTT results (Figure 2a). Thus, an increase in cell viability was observed over time. Additionally, no statistical differences were determined in groups, as compared to the control group (without melatonin group) on all days, which means that none of the melatonin doses had toxic effects on RAW 264.7 cells. Moreover, an increase in the mitochondrial activity was observed in groups containing 100 (P < 0.001), 250 (P < 0.001), and 500 µM (P < 0.01) melatonin concentrations on the last day of culture, indicating that melatonin can also increase the proliferation of precursor cells at these concentrations.

### 3.3. Composition of osteoclastogenic medium

According to the cell viability results given in Figure 2b, the statistical differences between cells cultured in proliferation medium (control group) and cells cultured in osteoclastogenic media containing either RANKL (R) or both RANKL and MCSF (RM) were not significant at days 3 and 7. However, at day 5 cell viability values were less than the control values (P < 0.01). This may be the result of the decrease in mitochondrial activity among cells during differentiation to osteoclasts. The TRAP enzyme activity that is responsible for the resorptive activity of osteoclasts was also determined. As seen in Figure 2c, TRAP activity was statistically higher in R and RM groups than in control on days 5 and 7. Thus, the statistical difference between control group and the other two groups was P < 0.01 for day 5 and P *<* 0.001 for day 7. However, no statistical difference was detected between R and RM groups on days 5 and 7.

**Figure 3:**
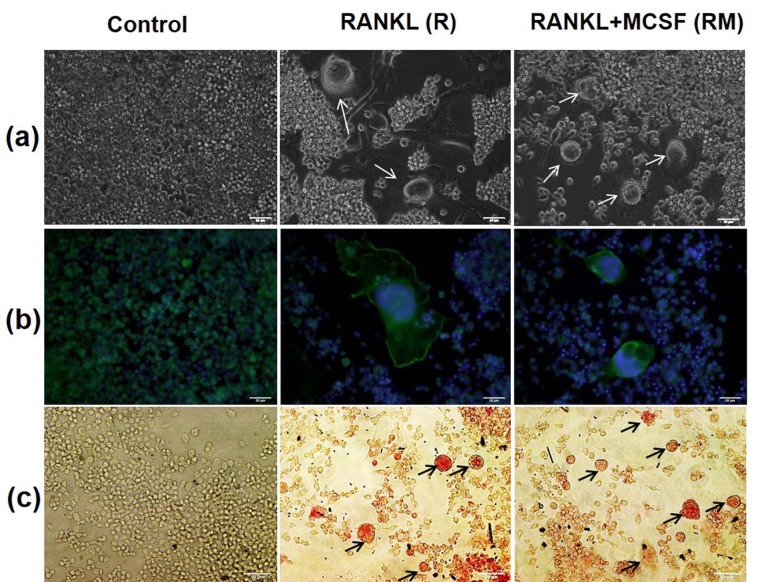
Morphological analysis of RAW 264.7 cells grown in different media at 32 X magnification (a) optic microscope images on day 6 (osteoclasts were marked with white arrows), (b) F-actin/DAPI staining on day 7 (cytoskeletons were colored with green while nuclei were colored in blue), (c) TRAP staining on day 5 (TRAP+ cells were shown with black arrows). Scale bar is 50 μm. (Control: cells in proliferation medium; RANKL: cells in proliferation medium containing 10 ng/mL RANKL; RANKL+MCSF: cells in proliferation medium containing 10 ng/mL RANKL and 10 ng/mL MCSF).

The morphological changes of RAW 264.7 cells before and after differentiation were observed by optic microscope, DAPI/F-actin staining, and TRAP staining over the culture period. Bigger and multinucleated cells were observed markedly by day 6 in R and RM groups and were not observed in the control group (Figure 3a). In addition, Figure 3b shows that differentiated cells are bigger than the undifferentiated ones. Additionally, the number of nuclei detected in differentiated cells was greater than 3, while undifferentiated cells had only one nucleus. The formation of TRAP+ cells was observed in the image field of RAW 264.7 cells stained with TRAP on day 5. As observed in Figure 3c, no TRAP+ cells were detected in the control group; however, TRAP+ cells were detected in R and RM groups, and there were more of these cells in RM group than in R group.

**Figure 4:**
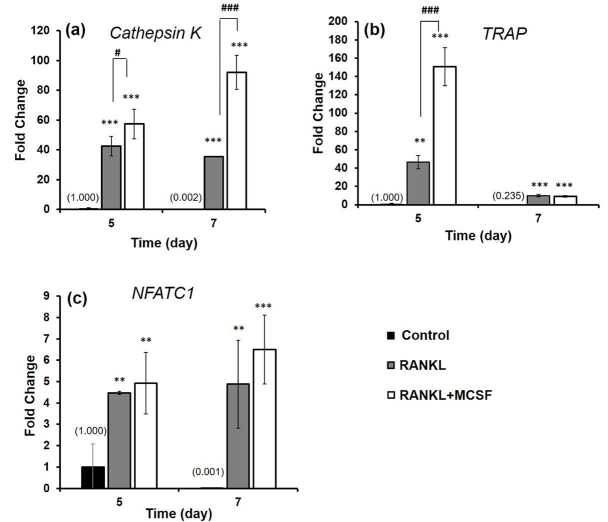
a) Cathepsin K, b) TRAP, and c) NFATC1 gene expressions of osteoclast marker genes on RAW 264.7 cells grown in proliferation and in differentiation media. (Statistical differences according to control group (cells grown in proliferation media): * P < 0.05, **P < 0.01, *** P < 0.001, to cells grown in proliferation media containing 10 ng/mL RANKL: # P < 0.05, ## P < 0.01, ### P < 0.001).

As seen in Figure 4a, expression levels of the cathepsin K gene on days 5 and 7 increased significantly (P < 0.001) in both R and RM groups, as compared to control. Moreover, the expression level of the cathepsin K gene was higher in RM group than in R group on days 5 (P < 0.05) and 7 (P < 0.001). Additionally, statistical differences were detected in TRAP gene expression within R (P < 0.01) and RM (P < 0.001) groups, as compared to control (Figure 4b). Even statistical differences were observed in R (P < 0.001) and RM (P < 0.001) groups compared to control on day 7, and expression levels decreased significantly when compared to the levels detected on day 5. Similarly, NFATC1 gene expression was higher in R and RM than in control; however, no statistical difference was detected between R and RM (Figure 4c). In conclusion, all gene expression levels in R and RM were statistically higher than those of control. In addition, gene expression levels in RM were relatively higher than those in R.

### 3.4. Inhibitory dose of melatonin

**Figure 5:**
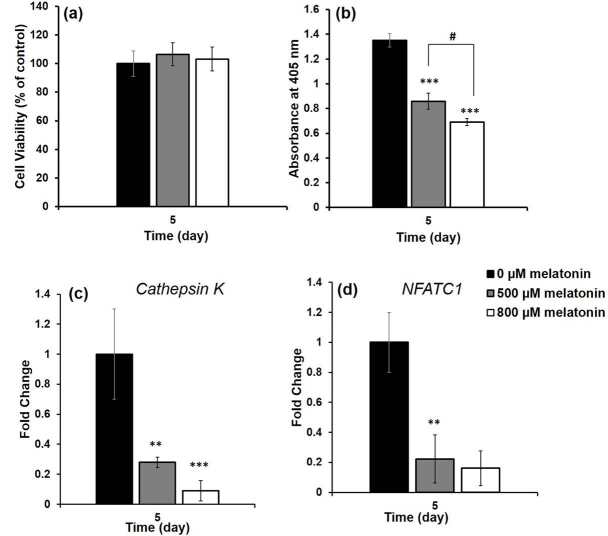
Viability and differentiation of RAW 264.7 cells grown in osteoclastogenic media containing 0, 500, 800 μM melatonin: (a) MTT results and (b) TRAP activity assay. (Statistical differences when melatonin-free group is control: * P < 0.05, ** P < 0.01, *** P < 0.001, when 500 μM melatonin containing group is control: # P < 0.05, ## P < 0.01, ### P < 0.001); c) Cathepsin K and d) NFATC1 gene expressions of RAW 264.7 cells grown in osteoclastogenic media containing different concentrations of melatonin. (Statistical differences when melatonin-free group is control: * P < 0.05, ** P < 0.01, *** P < 0.001).

**Figure 6:**
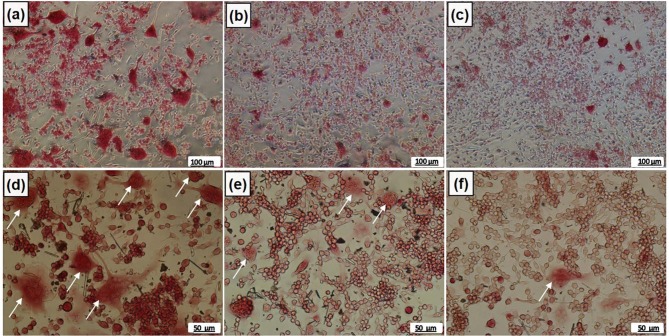
TRAP staining of RAW 264.7 cells in osteoclastogenic media containing (a) 0 μM MEL, (b) 500 μM MEL, (c) 800 μM MEL at 20 X magnification (scale bars represent 100 μm); (d) 0 μM MEL, (e) 500 μM MEL, (f) 800 μM MEL at 40 X magnification (scale bars represent 50 μm). TRAP+ cells with at least three nuclei were considered osteoclasts (white arrows).

Upon determination of melatonin cytotoxicity on the proliferation of cells and determination of the components of osteoclastogenic medium, two nontoxic doses of melatonin (500 and 800 µM) were selected. The experiments were conducted using 0, 500, and 800 µM melatonin containing osteoclastogenic media. The cells maintained their viability in all of the osteoclastogenic media at almost the same level; no statistical differences were detected among the groups (Figure 5a). TRAP activity was also measured and compared within the groups (Figure 5b). Absorbance values indicates that TRAP activity in groups containing both 500 µM and 800 µM of melatonin decreased significantly when compared to the melatonin-free group; thus, a significant statistical difference was determined (P < 0.001). In addition, there was a significant statistical difference between the 500 µM and 800 µM melatonin-containing groups (P < 0.05), suggesting that most of the inhibition occurred in the 800 µM melatonin-containing group. The relative expressions of osteoclast marker genes decreased markedly in osteoclastogenic media containing melatonin (Figures 5c and 5d). Statistical differences with respect to the melatonin-free group and cathepsin K gene expression in 500 µM and 800 µM melatonin-containing groups was P < 0.01 and P < 0.001, respectively, implying that the decrease in expression level in the 800 µM melatonin-containing group was more significant. In addition, the statistical difference according to control in NFATC1 gene expression for both melatonin-containing groups was significant (P < 0.01). TRAP+ cells having at least three nuclei were considered osteoclasts and stained with a claret-red color (Figure 6). The number of TRAP+ cells decreased in a dose-dependent manner in MEL; their number in the 800 µM melatonin-containing medium was lowest, suggesting that the inhibition of osteoclast formation increases with an increase in melatonin concentration.

## 4. Discussion

Melatonin takes part in several biochemical events involved in the bone repair process. For instance, the antioxidant properties of melatonin appear to have importance in the inflammatory stage, as melatonin can eliminate free radicals which may result in accelerating bone formation. Other studies have demonstrated that melatonin increases angiogenesis during bone repair, and this can also speed up bone formation (Liu et al., 2013). In addition, melatonin regulates the last stage (bone remodeling) in bone repair. Melatonin promotes osteoblastic differentiation via BMP/extracellular signal-regulated kinase (ERK)/Wnt pathways (Park et al., 2011). The OPG subsequently released from activated osteoblasts antagonizes RANKL; in this way, melatonin indirectly inhibits osteoclast differentiation (Koyama et al., 2002). In addition, previous studies have explained the dose-dependent diminishment of MEL in osteoclast differentiation by investigating the molecular basis of this process. The production of p65, the pivotal factor of NF-κB, decreased when it interacted with MEL, indicating that NF-κB signaling was suppressed by MEL (Ping et al., 2017; Zhou et al., 2017). An increase in osteoclast activity may lead to the development of bone diseases such as osteoporosis. Many antiresorptive therapies, including bisphosphonates, were found to reduce osteoclast formation and activity; however, side effects correlated with long-term administration, such as osteonecrosis and esophageal irritation, as well as their high cost, limit their use (Liu et al., 2013; Zhou et al., 2017). On the other hand, low cost and an absence of side effects made melatonin a good therapeutic agent for osteoporosis (Liu et al., 2013).

Most studies in the literature have used BMM cells to study the effect of MEL on osteoclastogenesis. For instance, the inhibitory effect of melatonin doses in the range of 1–1000 µM was investigated in BMM cells cultured in the presence of RANKL and MCSF concentrations greater than 10 ng/mL, and the generation of TRAP+ cells decreased proportionally with MEL concentration (Ping et al., 2017; Zhou et al., 2017). However, as mentioned earlier, RAW 264.7 cells are a better tool for studying osteoclastogenesis. One study found that a 100 µM melatonin concentration did not have a suppressive effect on the differentiation of RAW 264.7 cells to osteoclasts (Satue et al., 2015). Nevertheless, the inhibitory effect of melatonin doses greater than 100 µM on differentiation of RAW 264.7 cells was not reported in the literature, and the addition of such melatonin doses was not studied in conjunction with low concentrations of RANKL and MCSF. In our study, the effect of 500 µM and 800 µM concentrations of melatonin on the differentiation of RANKL-induced RAW 264.7 cells was investigated through the direct addition of melatonin to osteoclastogenic medium containing low concentrations of RANKL and MCSF (10 ng/mL).

We initially investigated proliferation and differentiation behavior of the cells and their interaction with MEL. First, the cells were characterized, and doubling time was calculated at 15 h which matches the value given by the ATCC. The heterogeneous morphology of cells was also determined by SEM. Subsequently, a range of melatonin doses was selected, and the effect of such doses was investigated through the behavior of cells grown in proliferation medium in order to determine whether growth inhibition resulted from the cytotoxic effect of melatonin on osteoclast precursors or from its inhibitory effect on osteoclast differentiation. It was found that melatonin did not produce cytotoxic effects on the cells in the range of 100–1000 μM, and that coincides with results in the literature (Phiphatwatcharaded et al., 2014).

The differentiation of RAW 264.7 cells to osteoclasts is mediated mainly by RANKL. The binding between RANKL and its receptor on osteoclast precursors begins their differentiation toward osteoclasts. While many studies in the literature demonstrated that the use of different RANKL concentrations (10–100 ng/mL) (Itoh et al., 2001; Ichikawa et al., 2006; Xu et al., 2009) caused the formation of TRAP+ cells, other research showed that RANKL effectively promoted the formation of multinucleated cells at 10ng/mL and did not increase their total number at higher concentrations, suggesting that osteoclast formation does not depend on RANKL concentration (Nguyen and Nohe, 2017). Furthermore, MCSF can enhance the RANKL-mediated osteoclastic bone resorption as it promotes the survival and motility of the osteoclasts formed (Hodge et al., 2011). However, most studies have used concentrations greater than 10 ng/mL; for instance, concentrations in the range of 25–100 ng/mL have been added to media supplemented with RANKL (Detsch et al., 2016; Germaini et al., 2017; Kim et al., 2017). In the present study, minimum concentrations of both factors were selected and the effect of RANKL, alone or in combination with MCSF, on the differentiation of cells was investigated. TRAP activity and relative expressions of osteoclast marker genes were higher in 10 ng/mL RANKL- and 10 ng/mL MCSF-containing medium than in 10 ng/mL RANKL-containing medium alone. Similarly, TRAP+ cell numbers were higher in 10 ng/mL RANKL and 10 ng/mL MCSF, indicating that using minimal concentrations of both RANKL and MCSF was more suitable for osteoclast differentiation than using RANKL alone.

In conclusion, osteoclastogenic medium containing relatively lower concentrations of RANKL and MCSF (10 ng/mL RANKL and 10 ng/mL MCSF) than reported in literature is appropriate for osteoclast differentiation, and it is cost-effective. The differentiation ability of the cells decreased in a dose-dependent manner, and 800 µM of melatonin had a better inhibitory effect on cell differentiation toward osteoclasts. The results demonstrated that the melatonin concentration selected may be used in the treatment of bone diseases, particularly when osteoclast activation is life threatening.
